# Unusual intraoperative finding of abnormal retroperitoneal ileum: a case report

**DOI:** 10.1093/jscr/rjz330

**Published:** 2020-01-13

**Authors:** Johanna C F Willburger, Daniel C Steinemann, Markus Von Flüe, Marc-Olivier Guenin

**Affiliations:** Department of Visceral Surgery, University Centre for Gastrointestinal and Liver Diseases, St. Clara Hospital and University Hospital Basel, Clarunis, Basel, Switzerland

**Keywords:** anatomy, embryology, sigma resection, retroperitoneum, surgery

## Abstract

A 52-year-old female patient diagnosed with an adenocarcinoma of the sigmoid colon underwent anterior resection with direct anastomosis. Intraoperatively, we found the ileum completely retroperitonealized. Previously, the patient was asymptomatic and no congenital syndromes were diagnosed. The intraoperative finding of abnormal anatomy made the mobilization of the left hemicolon and the vessel ligation more challenging. This anatomical situation is a rare variation due to an embryonic malrotation, which occurs in about 1:500 newborns.

## INTRODUCTION

The regular anatomic position of the ileum is intraperitoneal [1]. A retroperitoneal ileum has been described in rare cases. It may occur in case of malrotation (1:500), which is often associated with intestinal malformation or disorders such as Hirschsprung’s disease [2]. There are different degrees of malrotation. During the embryological abdominal development, the gut is supposed to turn 270° counterclockwise outside of the abdominal cavity and to return into the abdomen in the anatomical correct position. Depending on the time of defective development, there could be an incomplete or even nonrotation [3]. The Ladd’s bands and other congenital bands are tissue of the peritoneum that position and fixate the organs at the anatomical correct position [4].

Due to the different degrees of malrotation, only about 1:6000 patients are symptomatic [5]. Therefore, the incidence of retroperitoneal ileum might be underestimated considering the condition to be asymptomatic in most cases.

Knowledge of anatomic variation is important in abdominal oncologic surgery to obtain a clear exposition of the surgical anatomy for central vascular ligation and lymphadenectomy [6].

## CASE REPORT

We present the case of a 52-year-old female patient who was diagnosed with a malign polyp in the sigmoid colon after a screening colonoscopy in December 2018. The patient was asymptomatic without history of past intestinal symptoms or abdominal surgery. In the pathological analysis of the endoscopy, a moderately differentiated microsatellite stable tubular adenocarcinoma with a high-grade dysplasia as well as haemangiosis and lymphangiosis carcinomatosa was found and resected completely (pT1 L1 V1 Pn0 R0).

Due to the prognostic risk factors with haemangiosis and lymphangiosis carcinomatosa, a laparoscopic anterior resection was decided after discussion in the interdisciplinary tumor conference. The pre-operative computed tomography (CT) scan of the abdomen and thorax showed no sign of metastasis, and the colon and ileum had no suspect findings.

**Figure 1:**
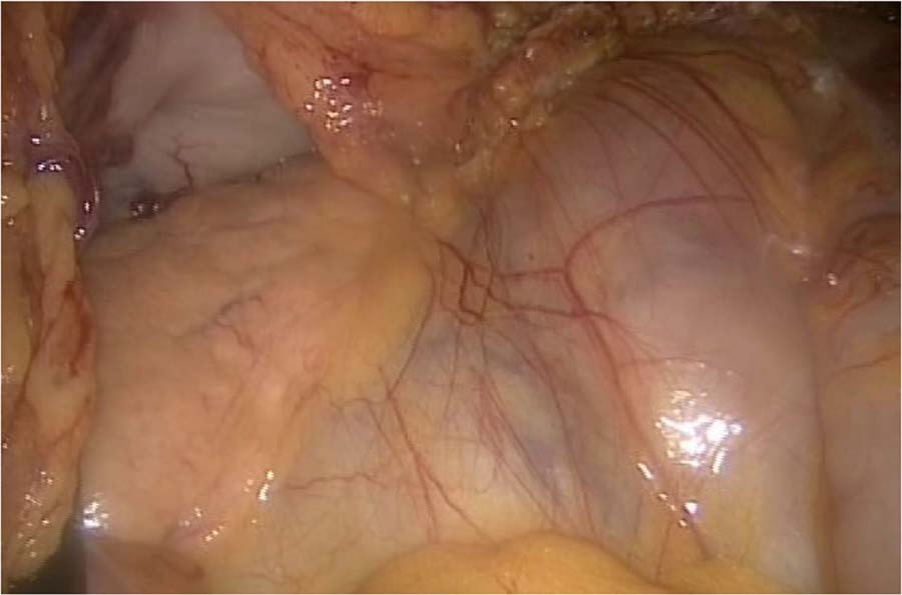
On the left side, we show the retroperitoneal pancreas, with the (as well) retroperitoneal jejunum on the right side.

**Figure 2:**
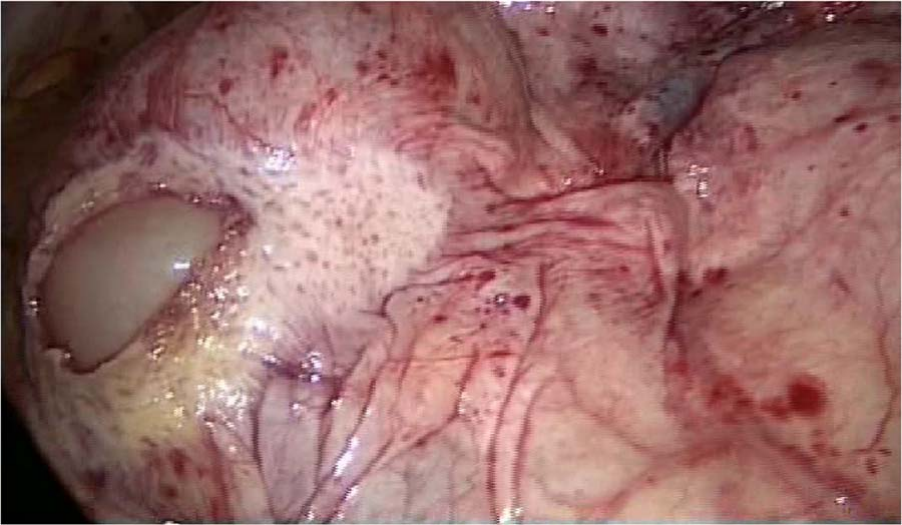
Dorsal peritoneum covering the entire ileum.

**Figure 3:**
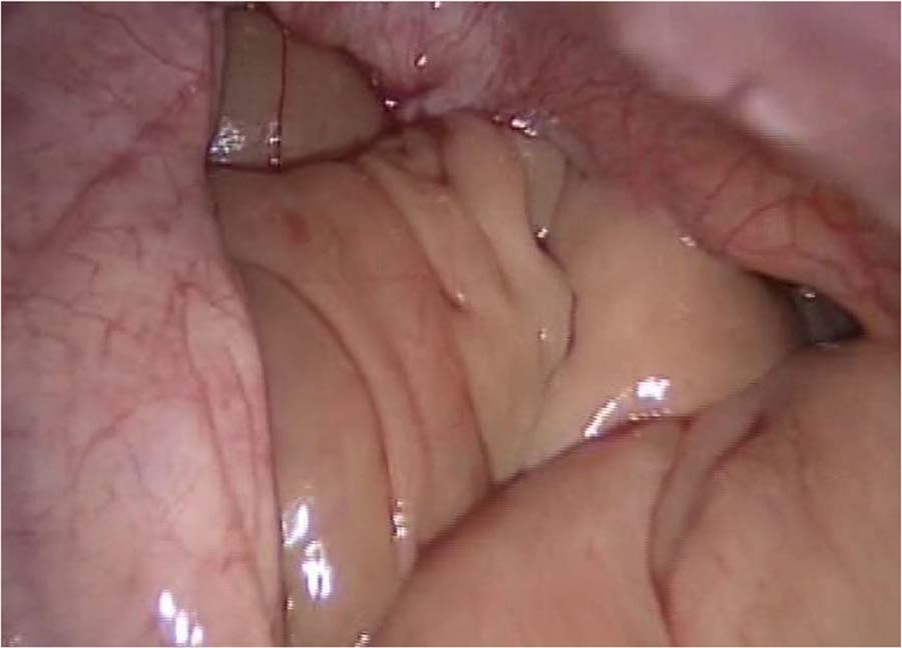
Retroperitoneal view, showing the ileum.

**Figure 4:**
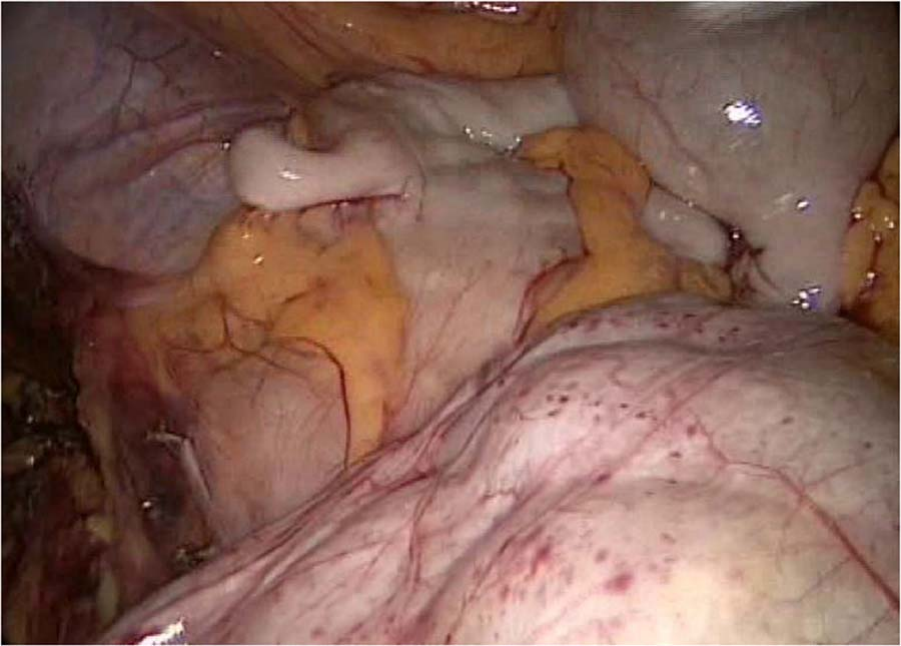
The slim intraperitoneal appendix in the left upper corner with the covered terminal ileum. In the right lower corner, the covered ileum convulse is seen.

The laparoscopic anterior resection was performed in February 2019. The patient was positioned in a modified lithotomy position. The pneumoperitoneum was established by a Veress needle. The trocars were placed umbilical, two on the left and one on the right abdominal wall. The descending colon and the endoscopic ink-marked region were visualized perfectly. While getting an overview of the abdominal cavity, we noticed an abnormality in the ileum region. It seemed that the peritoneum was covering the entire ileum. The situs is depicted in Figs 1–4.

The entire small bowel was found retroperitoneal covered by a slim peritoneal layer (Fig. 1). The dorsal peritoneum covering the ileum was opened (Fig. 2).

We accidentally opened the dorsal peritoneum (Fig. 2), and found the small bowel underneath - retroperitoneal (Fig. 3).

Even the terminal ileum seemed to be retroperitoneal. The slim preperitoneal appendix and the terminal retroperitoneal ileum are demonstrated in Fig. 4. The entire ileum was covered by the peritoneum.

We mobilized the left colonic flexure, descending and sigmoid colon as well as the proximal rectum. The visualization of the inferior mesenteric artery was impeded by the abnormal retroperitoneal position of the ileum. Therefore, we decided to perform a large (15 cm) Pfannenstil incision getting a better overview. Now we were able to identify the branches of the inferior mesenteric artery to follow those to the proximal trunk of the artery by mobilizing the ileum. After being sure about the anatomy, the vessel was dissected at the proximal origin using an endoscopic linear stapler. The proximal rectum was divided below the promontory and exteriorized through the Pfannenstil incision. The endoscopical ink-marked tumor position was localized and the tumor was resected. The minilaparotomy was closed, and after re-establishment of the pneumoperitoneum, a side-to-end transrectal descendorectostomy was fashioned using a circular stapler.

In the postoperative pathological analysis, there was a tubular adenoma with low grade dysplasia, which was resected in toto and one (1/22) lymph node metastasis from the previous known microsatellite stable tubular adenocarcinoma without perinodal infiltration of the soft tissue. The final TNM was pT1 pN1a (1/22) cM0 L1 V1 Pn0 R0; UICC stage IIIA [7].

In the interdisciplinary tumor conference, we suggested an adjuvant chemotherapy. The further clinical course was uneventful.

## DISCUSSION

A retroperitoneal ileum is a very rare finding, sometimes associated with other intestinal and embryonic abnormalities [8]. In our case, the whole ileum was retroperitoneal and the patient remained asymptomatic. In some cases, malrotation can cause symptoms or complications due to compression or a volvulus thus necessitating surgery. Furthermore, malrotation may be associated with chronic pain, nausea and vomiting, and obstruction. The diagnostic follow-up may be difficult especially in adults when the association with a congenital malrotation is not supposed and therefore adequate treatment may be retarded [4].

In the current case, not even the pre-operative CT scan was helpful detecting the retroperitoneal ileum, because the peritoneum cannot be visualized. Additionally, our patient did not have any further abnormalities of the gut or vessel variations that might have been suspicious. In these cases, we started surgery without being aware of the present condition that may represent a significant intraoperative challenge [9]. Therefore, it is important to know normal anatomy and embryological variations like malrotation [3], congenital adhesion bands (Ladd-bands), or position variations [4]. Furthermore, a malrotation is often associated with an abnormal anatomy of the abdominal vessels because the embryological rotation of the gut is supposed to be around the axis of the mesenteric vessels [3, 10]. Regardless our patient had no further anatomical variations, we needed to convert with a Pfannenstil approach to identify the course of the inferior mesenteric artery.

These variations must be recognized during surgery when to identify the important structures. If the surgeon is not aware of the anatomical structures, it should be converted to open surgery before passing the point of no return. In our case, the point of no return was the ligation of the inferior mesenteric artery so we decided to convert to a large Pfannenstil approach.

## CONCLUSION

In our presented case, we showed an asymptomatic anatomic abnormality, which was unable to be diagnosed preoperatively. That kind of situation could bring a surgeon in trouble if he/she cannot get an anatomical overview before resecting important structures. It is important to discover the situation if needed with an extra approach, as we did with the Pfannenstils approach.

## Conflict of interest statement

None.
